# The Territorial Army and Camp Sanitation

**Published:** 1908-08-08

**Authors:** 


### The Royal Army Medical Corps Section.

### THE TERRITORIAL ARMY AND CAMP SANITATION.

The first camp training under the new Territorial scheme is now in active progress, and throughout the United Kingdom there are under arms all over the country numerous infantry brigades (each of four battalions), as well as some Yeomanry regiments, many Field and Garrison brigades, and a large number of Army Service, Medical, and Engineer units. With the assemblage of such large bodies of men together in tents the preservation of their health under conditions entirely new and not altogether free from dangers becomes a serious responsibility, and throws on the medical officers of the units, as well as those in medical charge of the camps, very onerous duties. At the same time, however, the situation affords opportunities to medical officers of acquiring a knowledge of field sanitation, to which we would call special attention and of which we hope full advantage will be taken.

As is well known, of recent years the military authorities have laid great stress on the study of sanitation in the Regular Army, and have cordially supported the efforts of its medical officers in that direction. The result is that very steady and satisfactory progress has been made, but there is still ample room for improvements in many of the details of camp sanitation. No doubt the annual camping-out of units is primarily to perfect the men in their military duties, but what we would impress on the medical officers attending these camps is that there is also given them the means of studying, scientifically and practically, the conditions that are best suited for the maintenance of health in the field, and of which they should take the utmost advantage. Seeing, too, that camps are held in different localities, and consequently under conditions and surroundings that may vary widely, it follows that each camp has an importance that cannot be over-estimated, and one that extends to the sanitary report upon it. Useful, however, as are these sanitary reports from the medical officer in charge of the camp, we are of opinion that their value would be enhanced if they were accompanied in each case by similar reports from the medical officers of units embodying their views, experience, and suggestions. In this way we feel certain there would be brought together an amount of information and of useful hints that would prove of great service.

Medical officers will not be long resident in camp before they will realise that, apart from the general considerations of soil and situation that have to be taken into account in deciding on a camp site, the management and daily routine of camp life furnish many problems that are intimately bound up with the health of the inmates, and that call for ingenuity and resource to deal with them. Into these we cannot enter just now. What we would emphasise is the fact that in the main disease is preventable, and that in camp life the important factors that make for health are purity of water, good and well-cooked food, and the proper disposal of excreta and camp refuse. It is to these points special attention should be directed and clear definite orders issued in reference to them. But no orders are of use unless carried out efficiently, and the amount of sanitary knowledge possessed by the rank and file of the Territorial Army is not yet sufficient to allow of their being trusted to carry out these instructions. Consequently it is imperative to have some means of enforcing them, and the system of Camp Sanitary Sections seems the best method of ensuring the hygiene and sanitation of camps, especially if to some members of these sections are delegated the powers of sanitary police. Each Sanitary Section should consist of a sergeant and eight men, and should be made up of the pioneers of the battalion or of special men furnished by the various companies of the unit. This latter plan is preferable and has much to recommend it. By the aid of these Sanitary Sections excellent results can be attained.

Medical officers of units must, however, keep before themselves a higher ideal than sanitation by compulsion. They should aim at attaining it by hearty co-operation on the part of the regiments to which they are attached. This can only be done by spreading through all ranks from the commanding officer downwards, a knowledge of the laws of health and of the hygiene of camps, and by furnishing the *reasons* for the rules and regulations issued. If these latter are understood there will be a ready acceptance and due fulfilment of them. No better opportunity for such instruction could be afforded than the period of camp training, and medical officers should, by means of demonstrations and in other ways, enlist the interest and sympathy of the combatant officers in a branch of knowledge which is so intimately bound up with the welfare of the individual as well as with the success of every army in a campaign. The fact should never be lost sight of that at no period in our military history has the Medical Service of our Regular Army occupied a more respected position than it does at the present time. This very desirable state of matters is in no small measure due to the official recognition of the value and importance of army sanitation, and to the fact that the military authorities are now throwing on to the commanding officers the responsibility for the health of their respective units. The latter now see that their reputation and future advancement are to some extent bound up with the amount of illness amongst their men and the numbers fit for duty. Consequently, they are recognising in their regimental medical officers valuable advisers whose opinions they must seek and, to a large extent, follow. This is as it should be, and improves not only the status of the individual medical officers, but benefits indirectly the whole of the Territorial Medical Service.

It is very important that all the members of the Territorial Medical Service should realise this fact and do all they can to maintain the position, especially as everything is being done by the military authorities to help them to do so. The report just issued by the Director-General of the Army Medical Service on the progress made in constituting the Medical Service of the Territorial Force shows that the latter is placed on a most satisfactory and methodical basis. When completed it will include all the new formations calculated to secure efficiency, so that the medical arrangements of the Army for Home Defence will be furnished with field ambulances, sanitary companies, administrative medical officers, a Red Cross organisation for transporting the sick and wounded, general hospitals, and convalescent homes.

Admirable, however, as a system may be, it can only be a success if worked properly, and it is because we feel that the experiences gained in the annual camps may be very helpful in accomplishing this that we urge on all medical officers of the Territorial Army the importance of bringing thought and observation to their duties and embodying in individual reports all the experiences of their units. In this way they will make known many of the useful lessons they have learnt, and will enable the new Medical Service of the Territorial Army to contribute its quota of knowledge towards the solution of those many sanitary problems that are peculiar to military service.

